# Interaction of the Human Respiratory Syncytial Virus matrix protein with cellular adaptor protein complex 3 plays a critical role in trafficking

**DOI:** 10.1371/journal.pone.0184629

**Published:** 2017-10-13

**Authors:** Casey Ward, Maciej Maselko, Christopher Lupfer, Meagan Prescott, Manoj K. Pastey

**Affiliations:** Department of Veterinary Biomedical Sciences, Oregon State University, Corvallis, Oregon, United States of America; Kliniken der Stadt Köln gGmbH, GERMANY

## Abstract

Human Respiratory Syncytial Virus (HRSV) is a leading cause of bronchopneumonia in infants and the elderly. To date, knowledge of viral and host protein interactions within HRSV is limited and are critical areas of research. Here, we show that HRSV Matrix (M) protein interacts with the cellular adaptor protein complex 3 specifically via its medium subunit (AP-3Mu3A). This novel protein-protein interaction was first detected via yeast-two hybrid screen and was further confirmed in a mammalian system by immunofluorescence colocalization and co-immunoprecipitation. This novel interaction is further substantiated by the presence of a known tyrosine-based adaptor protein MU subunit sorting signal sequence, YXXФ: where Ф is a bulky hydrophobic residue, which is conserved across the related RSV M proteins. Analysis of point-mutated HRSV M derivatives indicated that AP-3Mu3A- mediated trafficking is contingent on the presence of the tyrosine residue within the YXXL sorting sequence at amino acids 197–200 of the M protein. AP-3Mu3A is up regulated at 24 hours post-infection in infected cells versus mock-infected HEp2 cells. Together, our data suggests that the AP-3 complex plays a critical role in the trafficking of HRSV proteins specifically matrix in epithelial cells. The results of this study add new insights and targets that may lead to the development of potential antivirals and attenuating mutations suitable for candidate vaccines in the future.

## Introduction

Human Respiratory Syncytial Virus (HRSV) is the leading cause of acute lower respiratory tract infection (ALRI) in infants and it is estimated that globally 66,000–199,000 children younger than 5 years died from HRSV-associated ALRI in 2005 [1]. HRSV belongs to the *Paramyxoviridae* family and is an enveloped, negative sense, single-stranded RNA virus encoding 11 proteins [2]. HRSV infection occurs preferentially through the apical surface of the most superficial layer of the polarized epithelium of the respiratory tract [3, 4]. Viral assembly and budding occur late in the viral life cycle at the apical surface and are coordinated by the interaction of the HRSV Matrix (M) protein with the cytoplasmic tails of viral proteins [2]. The envelope glycoproteins are translocated to lipid rafts at the apical membrane through an interaction with M along with the preformed nucleocapsids, facilitating final assembly and budding [2, 5].

The HRSV M protein exhibits a unique characteristic in that it localizes to the nucleus early in infection and exports late in infection. Thus, it has been proposed that the M protein may function as a type of ‘biological clock’ for the virus by entering the nucleus early in infection to inhibit host cell transcription, thereby focusing the efforts of the infected cells machinery on viral replication, then exiting late in infection from the nucleus to terminate viral replication in preparation for virus assembly, only once sufficient viral protein has accumulated in cells [2, 6, 7, 8].

There exists four heterotetrameric adaptor protein complexes, AP-1, AP-2, AP-3, and AP-4, which mediate the sorting of proteins to specific membrane components of the cell [9]. AP complexes are made up of two large subunits, a medium subunit, and a small subunit (delta and beta3, mu3 and sigma3 respective to the AP-3 complex). The AP-3 complex also contains two isoforms in its mu subunit, with mu3A expressed ubiquitously and mu3B expressed only in neurons and neuroendocrine cells [10]. The AP-3 complex specifically forms part of the cytoplasmic coat of vesicles that mediate transport of trans-membrane proteins between the *trans*-Golgi, endosomes and lysosomes. The selection of cargo by the AP complexes requires the recognition of specific motifs found in the cytoplasmic tail of transmembrane cargo [11]. These sorting signals include tyrosine-based motifs (YXXФ: where X can be polar residues and Ф is a bulky hydrophobic residue) and di-leucine/acidic residue-containing motifs [9, 10, 12, 13].

Here, we demonstrate that the AP-3 complex binds HRSV M via its mu-subunit and is further stabilized by an interaction with AP-3delta. In addition, point mutated derivatives of HRSV M in its conserved YXXL (Y, tyrosine; L, leucine) sorting motif at amino acids 197–200 (197-YXXL-200) impair the ability of HRSV M to traffic to proper sites of viral assembly late in infection.

## Materials and methods

### Screening of HeLa cDNA library with HRSV M using Matchmaker yeast two-hybrid system

The yeast two-hybrid screen was performed using Clontech’s Matchmaker two-hybrid system with a pre-transformed HeLa cDNA library as prey and the HRSV Matrix protein as bait. The RSV M/pAS2-1 plasmid was transformed into yeast strain PJ69-2A, and the transformed PJ69-2A cells were mated with a pre-transformed HeLa cell cDNA library (Clontech) in yeast strain Y187. Several novel interactions were detected using dropout medium lacking leucine, tryptophan, and histidine in addition to beta-galactosidase assays. Plasmids from positive interactions were isolated using the E.Z.N.A yeast plasmid kit (Omega) then sequenced at the Center for Genome Research and Biocomputing (CGRB) core facility at Oregon State University. Gene identity was determined by performing a BLAST search of the insert sequence.

### Yeast two-hybrid method to confirm specific interaction

The specificity of interaction of HRSV M and Adaptor protein 3 was further confirmed in yeast two-hybrid. Adaptor protein 1 and empty plasmids with out the cDNA fusions were used as a negative control. Fusions with the GAL4 DNA- binding domain (GAL4DB) were constructed in pGBT9, and those with the GAL4 activation domain (GAL4AD) were constructed in pGAD424 [14]. The cDNA fusions were performed by ligation of cDNA that had been amplified with the polymerase chain reaction (PCR) [15] using oligonucleotide linkers to allow the in-frame insertion into the yeast expression vectors. Sequencing of inserts was performed to make sure that the inserts have no mutations and are in frame with the GAL4DB/AD at Oregon State University’s Center for Genome Research and Biocomputing (CGRB) core facility.

### beta-Galactosidase assay

beta-Galactosidase activity in each of the transformants was measured. The colony beta-Galactosidase color assay and quantitative beta-Galactosidase assay were performed as described by Vojtek *et al*. [16]. Activity is expressed in standard units [1] multiplied by 1,000. All results were reproducible in at least two independent assays.

### Antibodies

The primary specific antibodies that were used are the following monoclonal mouse anti-Matrix (a kind gift from Erling Norrby and Mariethe Ehnlund, Karolinska Institute, Sweden), polyclonal goat anti-AP-3Mu3A (Santa Cruz Biotechnology, Cat# **sc-6425**), monoclonal mouse anti-AP-3delta (a kind gift from Andrew Peden, University of Cambridge), polyclonal goat anti-HRSV (Chemicon, Cat# AB1128), polyclonal goat anti-H1N1 (Thermo Fisher Scientific, Cat# PA1-7221), Mouse mAb anti-GAPDH (Calbiochem, Cat# CB1001) 1:4000 and monoclonal mouse anti-HIV-1 p24 Gag (NIH AIDS Research & Reference Reagent Program).

The secondary specific antibodies that were used are the following: donkey anti-mouse Alexa-Fluor 546 (Invitrogen, cat# A10036), rabbit anti-goat Alexa-Fluor 488 (Thermo Fisher Scientific, Cat# A-11078), donkey anti-goat IR Dye 800 (Rockland, Cat# 605-745-125) and donkey anti-mouse IR Dye 700 (Rockland, Cat# 610-744-002).

### Cell culture and transfection

HEp-2 (American Type Culture Collection, ATCC) cells grown at 37°C and 5% CO_2_ in Dulbecco’s Modified Eagles Medium (DMEM) supplemented with 100U penicillin, 100μg/mL streptomycin, 0.25μg/mL amphotericin B and 10% fetal bovine serum (P/S/A/FBS) were transfected with indicated constructs using Fugene HD (Roche) according to plasmid optimization and manufacturer’s protocol.

### Virus and infection

The laboratory strain VR-1540^™^ HRSV A2 (American Type Culture Collection, ATCC) was grown, assayed and stored as previously described [15]. HEp2 cells were plated day before infection at desired confluency, HRSV was adsorbed for one hour with 10 minute interval shaking at indicated MOI at 37°C and 5% CO_2_ in DMEM + FBS. Virus was removed and replaced with DMEM + P/S/A/FBS. Virus stocks were stored at −80°C until used. Stock virus titers were determined by plaque assay.

### Plaque assay

HRSV plaque assays were performed in HEp-2 cells seeded into 24-well plates and grown until confluent. Serial tenfold dilution of samples was made and plaque assays performed as previously described [15]. Virus titer was determined by the highest dilution in which duplicate wells contained at least 5 plaques.

### Plasmid constructs

The construct used to express the HRSV M protein in mammalian cells was a codon optimized M protein (Opt.M) (a kind gift from James E. Crowe, Jr., Vanderbilt University School of Medicine). The Opt.M construct and point-mutated derivatives thereof were subsequently placed in-frame into pcDNA 3.1(-) (Invitrogen) and pEGFP-C1 (Clontech) vectors using *EcoR*I and *BamH*I restriction sites. These plasmid constructs were used for co-immunoprecipitation and colocalization studies respectively.

To generate the Y197A point-mutated Opt.M pcDNA 3.1(-) construct a specific forward primer (TCATCCCT**GC**CTCAGGATTACTATTAGTCATCACA [boldface type indicates Y197A point-mutation]) and a reverse primer complementary to Opt.M with a *BamH*I site (GAGCTCGGATCCTCAGTCCTC) [the underlined sequence indicates the *BamH*I restriction site] were used to introduce the Y197A point–mutation and amplify the desired regions of Opt.M (194–256 amino acids [aa]) with the Opt.M pcDNA 3.1(-) construct used as template. A specific reverse primer (ATCCTGAG**GC**AGGGATGATTTTTGCATTTGTGATA [boldface type indicates Y197A point-mutation]) and a Opt.M specific forward primer with a *EcoR*I site (TCTGCAGAATTCGCCACCATGGAGACC [the underlined sequence indicates the *EcoR*I restriction site]) were used to introduce the Y197A point-mutation and amplify the desired regions of Opt.M (1–199) aa). These initial overlapping PCR fragments were made using Pfx polymerase kit (Invitrogen). Aliquots from each of the above reactions were combined and used as template for the PCR ligation step to make the full length Y197A Opt.M DNA construct using PCR Supermix with Taq (Invitrogen).

The protocol described above was also used to generate the L200A Opt.M overlapping fragments and subsequent full length DNA construct with the following changes in the specific primers used to introduce the desired L200A point-mutation: specific forward primer (TACAGCGGC**GCC**CTGCTGGTGATC [boldface type indicates L200A point-mutation]) to introduce the L200A point-mutation and amplify the desired regions of Opt.M (197–256) aa and specific reverse primer (CACCAGCAG**GGC**GCCGCTGTAAGG [boldface type indicates L200A point-mutation]) to introduce the L200A point-mutation and amplify the desired regions of Opt.M (1–203) aa. Full-length constructs were placed into appropriate vector, screened and sequenced to confirm proper insertion at Oregon State University’s Center for Genome Research and Biocomputing (CGRB) core facility.

### Colocalization and immunofluorescence

Subconfluent (80%) HEp2 cell monolayers grown on glass coverslips were infected with HRSV A2, mock-infected or transfected with various plasmid cDNAs and cultured for the indicated times after infection or transfection. Cells were washed with ice-cold PBS and fixed with 4% paraformaldehyde for 10 min at room temperature, followed by the permeabilization of membranes with 0.2% Triton X-100 for 5 min. Fixed cells were washed thoroughly in PBS and incubated for one hour in bovine serum albumin (BSA)-PBS (1% BSA/PBS) as a preliminary blocking step. Subsequently, fixed cells were incubated for one hour in primary, specific antibody diluted in 1% BSA/PBS blocking buffer. Fixed cells were extensively washed with a Tween-20 containing wash buffer and bound antibodies were detected with species-specific fluorochrome-conjugated secondary antibodies via one-hour incubation in dark. Coverslips were mounted in fluorescent mounting medium containing DAPI (Vectashield, Vector Laboratories) and analyzed by confocal laser scanning microscopy (CLSM). Pinhole diameter was 1 μm for all analyses. Multi-track configuration was performed on CGRB Zeiss 540 Meta Confocal Microscope. Images of more than 20 cells were analyzed for each sample.

### Co-immunoprecipitation

Subconfluent (90%) HEp2 cell monolayers grown in a 75cm^2^ flask were either infected or mock-infected with HRSV A2 at an MOI of 5. Post 24 hours infection, 1 ml ice cold MPER (Pierce) buffer was added to cell monolayer and incubated for 10 minutes on a rocker. Cells were scraped and lysate was vortexed briefly in a microcentrifuge tube. Cellular debris was pelleted by centrifugation at 10,000xg for 10 minutes at 4°C. Supernatant was transferred to new tube together with 1.0 μg of specific antibody and incubated at 4°C for ≥1 hour. 20μl of resuspended volume of Protein A/G PLUS-Agarose (Santa Cruz Biotechnologies) was added and then further incubated at 4°C on rotating device overnight. Immunoprecipitates were collected by centrifugation at 2,500 rpm for 5 minutes at 4°C. Supernatant was then discarded and pellet was washed 4 times with 1.0 ml PBS, each time repeating centrifugation step above. After final wash, supernatant was aspirated and discarded and pellet was resuspended in 40μl of 1x LDS electrophoresis sample buffer (Invitrogen). Samples were boiled for 2–3 minutes (70°C) and 20μl aliquots were analyzed by SDS-PAGE Western Blot. Blots were transferred to nitrocellulose, blocked for one hour in (Odyssey) blocking buffer then probed with either goat-anti-HRSV polyclonal antibody or monoclonal mouse anti-HRSV M. Blots were washed four times for 5 minutes after each incubation with antibody in PBS-H_2_O with 0.2% Tween 20 washing solution, and then were probed with IR labeled specific secondary antibodies, washed, and analyzed on an Odyssey Infrared Imaging Center (Licor).

### Western blot and fluorescence intensity analysis

6-well plates were seeded with approximately 1.0 x 10^6^ HEp2 cells, mock-infected or infected next day at an MOI of 5, then proteins were extracted using MPER (Pierce) with Protease Arrest and scraping from triplicate wells at respective times post-infection. Cell lysates plus NuPAGE Reducing Agent (Invitrogen) were heated at 70°C for 10 minutes and equal amounts of protein were resolved on 4–12% Bis-Tris gels (Invitrogen) in triplicate, followed by transfer to nitrocellulose. Blots were blocked in Rockland IR blocking buffer overnight and then probed with Goat anti-Ap3Mu3A (1:200), Mouse anti-RSV Matrix (1:1000), Rabbit anti-delta adaptin (SA4) (1:1000), Rabbit anti-GAPDH (1:10000) for one hour. Washed extensively in Tween-20 containing wash buffer, then blotted with species-specific IR labeled antibodies: Donkey anti-Goat IR 800 (1:25000), Donkey anti-Rabbit IR 800 (1:25000), Donkey anti-Rabbit IR 700 (1:5000), Donkey anti-Mouse IR 700 (1:5000), Donkey anti-Mouse IR 800 (1:20000) for an hour and then washed extensively. Blots were imaged on Licor IR Imager, fluorescent intensity analysis was performed using Licor imaging program and subsequent data analysis was performed by comparing infected versus uninfected triplicate averages of the integral intensity ratio between GAPDH loading control and AP3 integral intensities via unpaired two tail t-test.

### Statistics

All statistics were performed using GraphPad Prism version 4.00 for Windows (GraphPad Software, San Diego, CA) by one-way ANOVA and either Bonferroni or Dunnett’ post-tests, or two-way ANOVA with Bonferroni post-test.

## Results

### HRSV M binds to the MU subunit of the AP-3 complex

We performed a yeast-two hybrid screen of a HeLa cDNA library using full-length HRSV Matrix as bait to identify Matrix binding partners. Our screen identified six candidate Matrix binding partners using dropout medium lacking leucine, tryptophan, and histidine in addition to beta-galactosidase assays. We chose one of these candidates for further evaluation, the AP-3Mu3A protein, based on its significant role in interactions with the matrix protein of other viruses. One other candidate, ubiquitin C was shown by our lab to be important in HRSV pathogenesis [15].

To test the specificity of interaction of HRSV M and AP-3Mu3A, we fused the GAL4DB with HRSV M and the GAL4AD with AP-3Mu3A. The GAL4AD with AP-1 and empty plasmids were used as negative controls. These plasmids were then co-expressed in *S*. *cerevisiae* Y153, and the intensity of the interaction was measured either in the colony color assay or as beta-Galactosidase activity (Table 1). Interaction between HRSV M and the AP-3Mu3A was the strongest, with a blue color being detectable within 2 h by colony filter color assay. Controls in which either the HRSV M or the AP-3Mu3A was omitted showed no blue color for up to 12 h (Table 1). Likewise, there was no interaction when HRSV M was paired with constructs containing the AP-1. Interaction between HRSV M and AP-3Mu3A was orientation independent as switching between GAL4DB and GAL4AD did not significantly alter beta-Galactosidase color assay units. The results show that the interaction between HRSV M and AP-3Mu3A was specific in yeast two-hybrid system.

**Table 1:** Interaction of adaptor proteins with the RSV matrix protein in a yeast two-hybrid system.

GAL4DB	GAL4AD	Colony color	beta-Galactosidase activity, units
Empty	Empty	---	1-2
Empty	Adaptor 3	---	1-2
HRSV M	Empty	---	1-2
HRSV M	Adaptor 3	+++	458
HRSV M	Adaptor 1	---	5-6
Adaptor 3	HRSV M	+++	420

The intensity of the interaction was measured either in the colony color assay or as beta-galactosidase activity. ‘‘Empty” indicates no insert in the vector. (*A*) Colony color assay. The colonies were grown on a plate containing appropriate medium, transferred onto a nitrocellulose filter, and lysed by rapid freezing in liquid nitrogen. The filters then were placed on a plate containing Z-buffer and 5-bromo-4-chloro-3-indolyl beta-D-galactoside (X-Gal) to analyze the enzyme activity. When HRSV M and Adaptor 3 were coexpressed in yeast, colonies showed a blue color within 2 h, whereas controls in which either the HRSV M or the Adaptor 3 were omitted showed no blue color for up to 12 h. (*B*) Quantitation of the interaction between HRSV M and the Adaptor 3 proteins. The colony color assay is graded as white (---) or dark blue (+++). beta-Galactosidase activity was quantitated and expressed in standard units multiplied by 1,000. The results were reproducible in at least two independent assays.

### HRSV M protein colocalizes with AP-3Mu3A protein in HRSV infected cells

**Fig 1:**
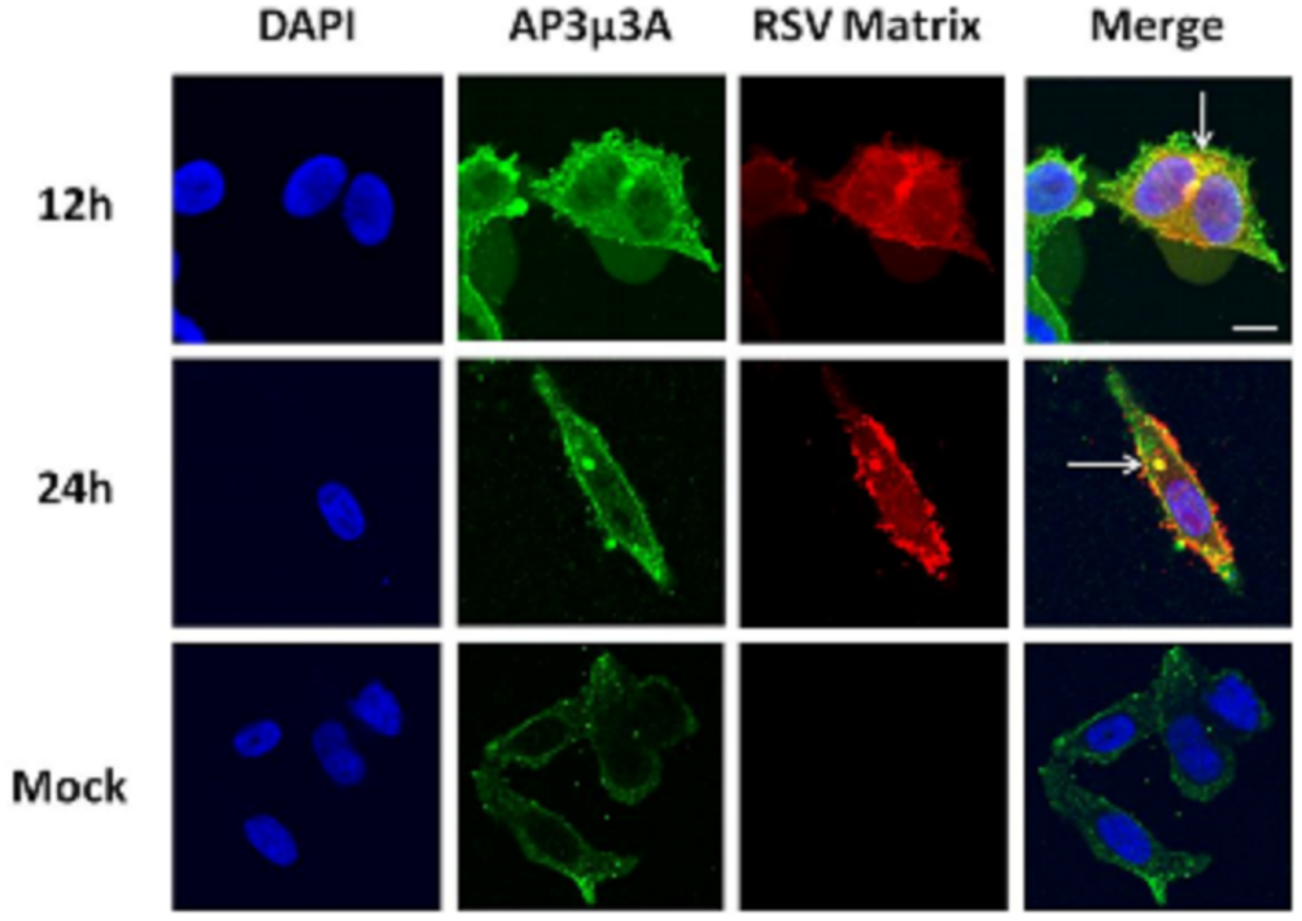
The M protein is seen associating with the AP-3Mu3A protein in distinct juxtanuclear cytoplasmic regions at 12 hours p.i. and in cytoplasmic inclusions at 24 hours p.i. HRSV-infected and mock-infected HEp2 cells were fixed 12 h and 24h after infection and were double stained with various antibody combinations, followed by CLSM analysis. The antibodies used are as indicated: goat anti-AP-3Mu3A (1:50), mouse anti-Matrix (1:100), rabbit anti-goat Alexa-Fluor 488 (1:200), donkey anti-mouse Alexa-Fluor 546 (1:200). Primary antibodies were incubated for 60 minutes; cells were then washed with Tween 20 wash solution and then subsequently incubated for 60 minutes with secondary antibodies. DAPI, is the 405 nm output corresponding to a blue color in the image above; AP-3Mu3A, is the 488nm output corresponding to the green color in the image above; HRSV Matrix, is the 546nm output corresponding to the red color in the image above; Merge, is the computer-generated merged image of all three outputs, with yellow coloration indicating colocalization. The results were reproducible in at least three independent assays. Scale bar is 10μm.

To investigate the association of the M protein with AP-3 in HRSV-infected cells, fixed HEp2 cells were probed with HRSVM and AP-3Mu3A specific antibodies and examined by confocal laser scanning microscopy (CLSM). At 12 hours post infection (p.i.), the M protein is localized at juxtanuclear regions of the cytoplasm with a small contingency still residing within the nucleus (Fig 1A). At 24 hours p.i., M is seen localizing to the cytoplasmic side of the membrane at sites of viral assembly and budding as well as in distinct cytoplasmic inclusions containing the viral nucleocapsid proteins (L, N, P, M2-1) that are sites of viral transcription [14] (Fig 1B). This expression pattern for M is consistent with previously published data [2, 7, 14]. The AP-3Mu3A protein complex localizes to the juxtanuclear region extending out through the cytoplasm to the cell margin at 12 hours post HRSV infection (Fig 1A). While at 24 hours, AP-3Mu3A is seen extending out through the cytoplasm to the cell margin as well; it is also localized within the cytoplasmic inclusions formed by the HRSV nucleocapsid proteins (Fig 1B). The cytoplasmic and juxtanuclear fluorescent pattern for the AP-3 adaptor complex is consistent with previously published data [17, 18, 19, 20, 21]. Colocalization of M with the AP-3Mu3A protein can be most distinctly observed in the juxtanuclear region at 12 hours p.i and in cytoplasmic inclusions at 24 hours. In both time points colocalization can be observed at various sites in the cytoplasm as well as at the cell margin (Fig 1A and 1B). The colocalization observed at 12 and 24 hours post HRSV infection suggests a direct interaction between the HRSV M and AP-3Mu3A and may represent a pathway for the association of the M protein and viral nucleocapsids late in HRSV infection. In addition, mock-infected HEp2 cells showed no bleed-through or background fluorescence and further validated the specificity of both AP-3Mu3A and M antibodies (Fig 1C).

### AP-3Mu3A and AP-3delta co-immunoprecipitate with the HRSV M protein

**Fig 2:**
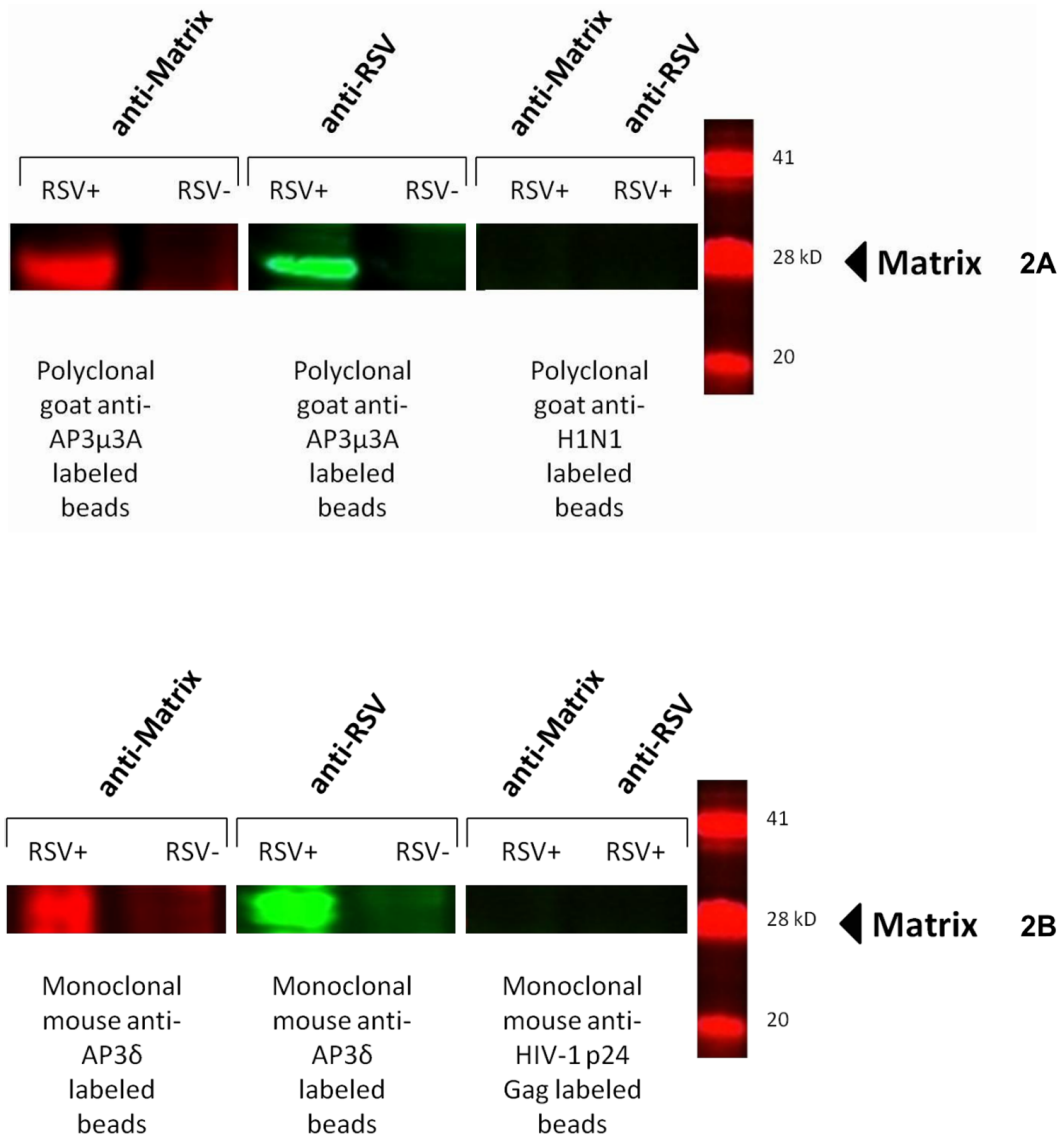
The HRSV M protein co-immunoprecipitates with the AP-3Mu3A and AP-3delta complex during HRSV infection. HEp2 cells at approximately 90% confluency were either infected at an MOI of 5 or mock infected for 24 hours, cells were scraped or proteins were subsequently extracted using MPER. Cell lysates were incubated for 6 hours with 1 μg of either polyclonal goat anti-AP-3Mu3A or monoclonal mouse anti-AP-3delta along with a antibody specific isotype control, polyclonal goat-anti H1N1or monoclonal mouse anti-HIV-1 p24 Gag at 4°C on a rotating device. 20μl Protein A/G agarose beads were added to lysate plus corresponding antibody and incubated overnight. Immunoprecipate complex was pelleted and washed with PBS and then ran out on a SDS-PAGE gel and transferred to nitrocellulose membrane. Membrane was blocked and then probed with either monoclonal mouse anti-Matrix or polyclonal goat-anti HRSV primary antibody as described previously for one hour. Membranes were then washed with a PBS-Tween20 solution extensively and then probed with species-specific secondary antibodies donkey anti-goat IR dye 800 and donkey anti-mouse IR dye 700. Membranes were again washed extensively and blots were imaged on Odyssey Infrared imager. The results were reproducible in at least two independent assays.

To further confirm the interaction between AP-3Mu3A and HRSV M and to determine if auxiliary stabilization existed from a corresponding subunit of the AP-3 hemicomplex, a co-immunoprecipitation analysis was performed. Immunoprecipitation of AP-3Mu3A and AP-3delta using an anti-AP-3Mu3A polyclonal and an anti-AP-3delta monoclonal antibody respectively, was carried out using cell lysates from both HRSV and mock infected HEp2 cells at 24 hours p.i. Through immunoblotting for HRSV proteins, both an anti-HRSV polyclonal and an anti-Matrix monoclonal antibody detected the 28kD Matrix protein in HRSV infected lanes alone (Fig 2A and 2B; S1 Fig). This further substantiates a protein-protein interaction between the AP-3Mu3A subunit and the HRSVM protein as well as indicates that at least the AP-3delta subunit of the AP-3 hemicomplex is involved in further stabilization of this interaction. To insure the specificity of both AP-3 antibodies in pulling down the M protein under these co-immunoprecipitation conditions and protocol, anti-H1N1 polyclonal and anti-HIV p24-GAG monoclonal antibodies were used as isotype controls to validate the results of AP-3Mu3A and AP-3delta experiments respectively. These control experiments resulted in no detection of HRSV M or other viral proteins suggesting that the precipitation of the HRSVM protein via the AP-3 complex is a specific interaction (Fig 2A and 2B; S1 Fig).

### Conservation of 197-YXXL-200 motif in related-RSV strains

**Fig 3:**
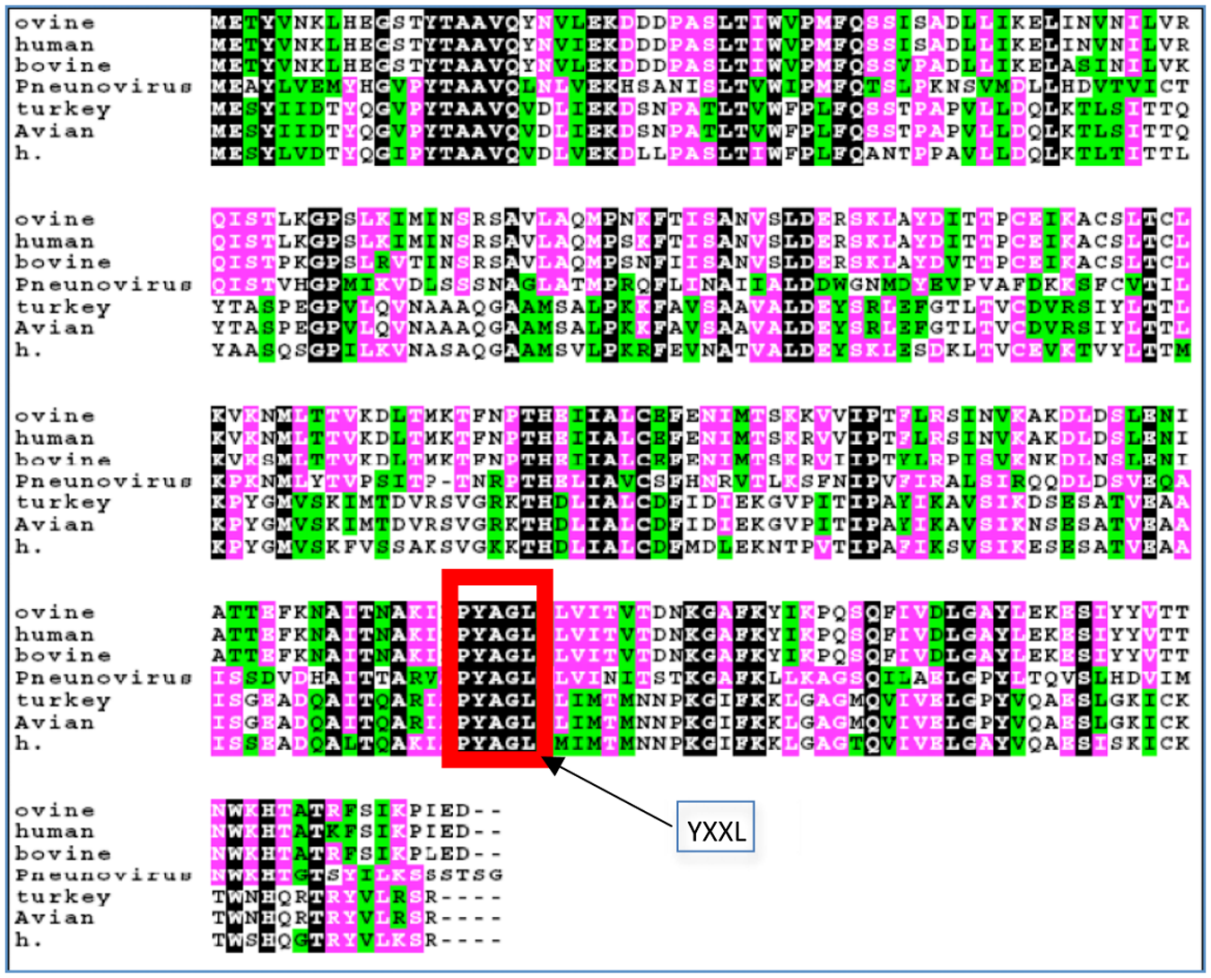
RSV matrix sequence alignment. ClustalW sequence alignment of Matrix protein comparing human RSV with various animal respiratory syncytial virus strains. The available sequences are aligned via ClustalW with color coding to indicate conservation (Black = identical, pink = strong similarity, green = weak similarity, white = no similarity). The conserved YXXL among human RSV is shown in a box.

To determine if an interaction between AP-3Mu3A and M is critical for RSV and to determine whether the motif is conserved among RSV strains that may play a role in maintaining pathogenesis and/or function, a sequence alignment was performed comparing the various RSV-related Matrix proteins as well as human metapneumovirus A (HMPV A). As shown in Fig 3, a clearly defined tyrosine based YXXL homologous region is retained at amino acids 197–200. In addition, there exists a subsequent YXXL region at amino acids 20–23 of the HRSV M protein, but this motif was not conserved across the same RSV-related strains and was thus not explored further in this study (data not shown). The presence of this highly conserved homologous region provides further evidence that an interaction with the AP-3Mu3A subunit is critical for RSV pathogenesis across various RSV-related strains.

### 197-YXXL-200 binding motif is critical for interaction of M protein with AP-3Mu3A

**Fig 4:**
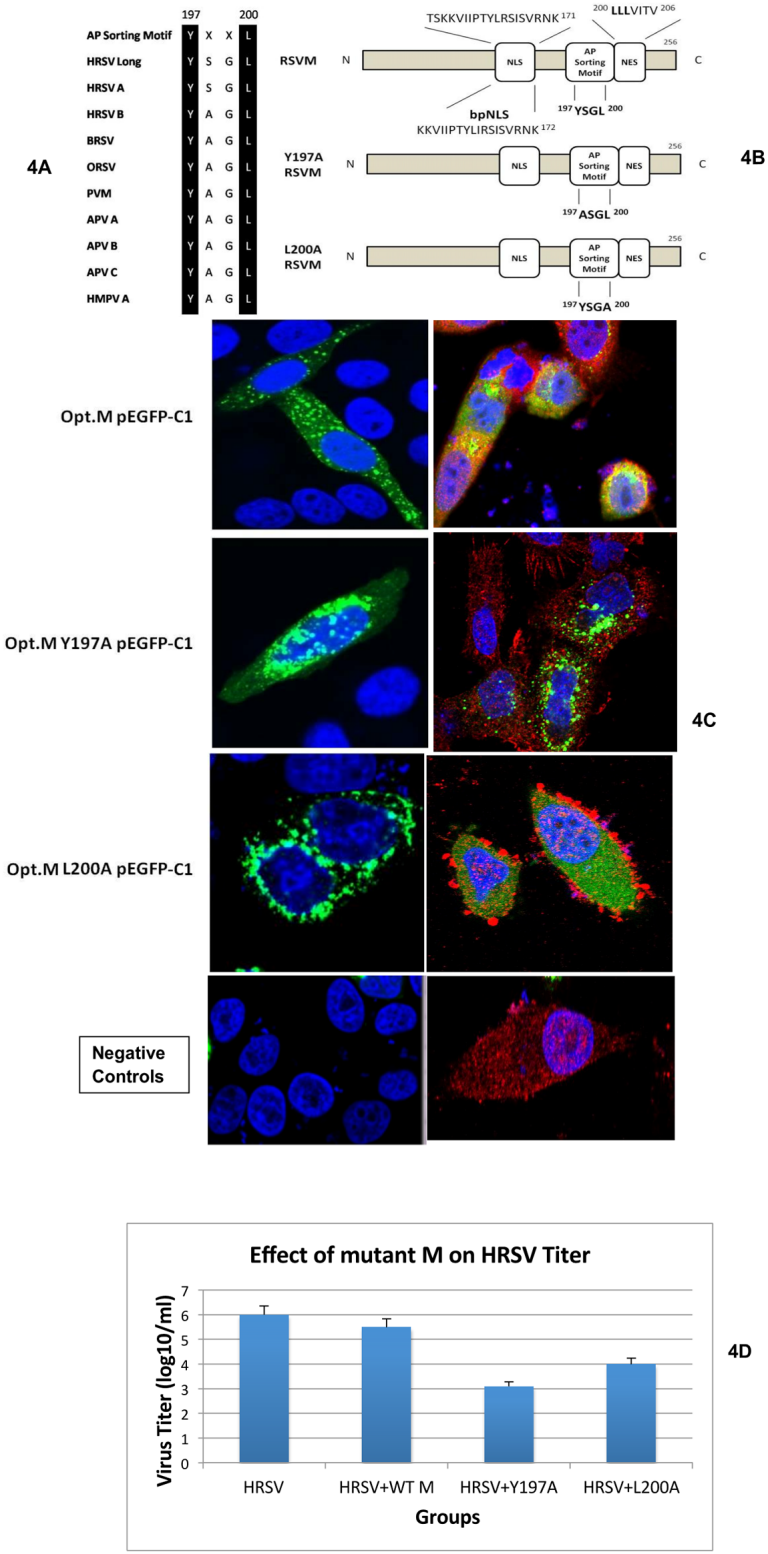
Effect of mutation in YXXL domain of HRSV M protein. **A**) Sequence alignment of Matrix protein amino acids 197–200 comparing various animal respiratory syncytial virus strains. The black regions highlight the conserved tyrosine and the Tyr+4 hydrophobic amino acid residue (leucine) at residues 197 and 200 respectively. [*Human HRSV Long Strain (HHRSV Long)*, *HHRSV Strain A*,*B (HHRSV A*,*B)*, *Bovine HRSV (BHRSV)*, *Ovine HRSV (OHRSV)*, *Pneumonia Virus of Mice (PVM)*, *Avian Metapneumovirus Strain A*,*B*,*C (APV A/B/C)*, *Human Metapneumovirus Strain A (HMPV A)*]. **B**) Schematic diagram of the Opt.M and point-mutated Opt.M constructs. The diagram highlights the proposed adaptor protein (AP) basolateral sorting motif (shown in single letter amino acid code), the putative nuclear export signal (NES) (8) and the nuclear localization signal within amino acids 110–183, the binding site for Importin beta1 (10). Single letter code values refer to the beginning of the M amino acid sequence. Bold letters indicate the putative 197-YXXL-200 and possible di-leucine sequence motif located in M from amino acids 200–202. **C**) The mutation of the tyrosine at amino acid residue 197 to alanine and leucine at amino acid residue 200 to alanine causes a distinct phenotypic change in the localization of the HRSV Matrix protein. HEp2 cells were transfected with indicated pEGFP-C1 Opt. M constructs and were fixed with paraformaldehyde at 24 hours post transfection as described previously. Cells were imaged for Opt.M, adaptor protein and DAPI using CLSM protocols described previously. Negative controls include transfection of cells with empty plasmid vector (pEGFP-C1) without M insert (left side panel) and mock transfected cells with staining for adaptor protein only with goat anti Ap3u3A followed by rabbit anti goat Alexa Fluor 546 antibody (right side panel). The first 3 images on the left side panel shows staining for Opt.M and DAPI. The first 3 images on the right side panel shows merged image with staining for Opt.M, adaptor protein and DAPI. The results were reproducible in at least two independent assays. **D**) Mutation in YXXL domain of M reduces HRSV titer. The HEp-2 cells were transfected with control plasmid, WT-M, Y197A or L200A mutant plasmids and infected with HRSV. Four days after infection, supernatants were collected, and the virus titer was measured by plaque assay. Representative data from three independent experiments are shown. The results were reproducible in at least two independent assays.

To confirm that the conserved 197-YXXL-200 sequence motif is essential for the proper interaction between M and AP3Mu3A, point mutations of the tyrosine and leucine residues of the 197-YXXL-200 sorting signal motif to alanine were designed, Y197A and L200A respectively (Fig 4A). We evaluated the phenotypic difference between the wt, Y197A and L200A Opt.M constructs via cloning into a fluorescence expression vector, pEGFP-C1. One-half of the cell culture wells from each transfected cell group (wt, Y197A and L200A) were infected with HRSV and were stained with primary polyclonal goat anti-HRSV antibodies (Chemicon) and secondary donkey anti-goat IR Dye 800 antibodies (Rockland). At 24 hours post-transfection, CLSM analysis showed the wt Opt. M construct localizing throughout the cytoplasm in a punctuate pattern, especially absent from the nucleus (Fig 4B). HRSV colocalizes with the wt M as indicated by the image with yellow coloration (Fig 4B). This expression pattern is consistent with the localization of M late in RSV infection. In comparison, the Y-197-A and L200A Opt.M constructs were seen localized exclusively in nuclear and juxtanuclear regions especially absent in cytoplasmic regions extending to the cell margin (Fig 4C). The HRSV may be seen budding but appear stuck and unable to get released from the cell membrane. The localization of the Y197A and L200A Opt.M mutant was similar to the expression pattern seen in HRSV infected cells 12 hours p.i. This observation suggests that because of the mutation of these vital tyrosine and leucine residues, the ability of AP-3 to direct M properly away from the juxtanuclear region toward cytoplamsic inclusions and/or sites of viral assembly late in infection was halted or impaired.

The supernatant collected from infected wells in each group at day 4-post infection was subjected to plaque assay for virus titer determination (Fig 4D). The viral titers from mutant M protein decreased at least by 2–3 logs compared to wt or HRSV alone. The HRSV produces its own matrix proteins and competes with the transfected mutant M protein and the reduction of viral titer may be due to mutant M protein incorporation into viral assembly and budding. The virus stuck on the cell membrane may not have been released into the media supernatant and this may account for reduction of viral titers in the mutant cell group. In order to get significant reduction of viral titers and the effect of virus infection, a recombinant HRSV with mutation of M in the viral genome is required which we plan to conduct future experiments with experts in the generation of recombinant HRSV.

### AP3Mu3A is specifically up-regulated late in HRSV infection

**Fig 5:**
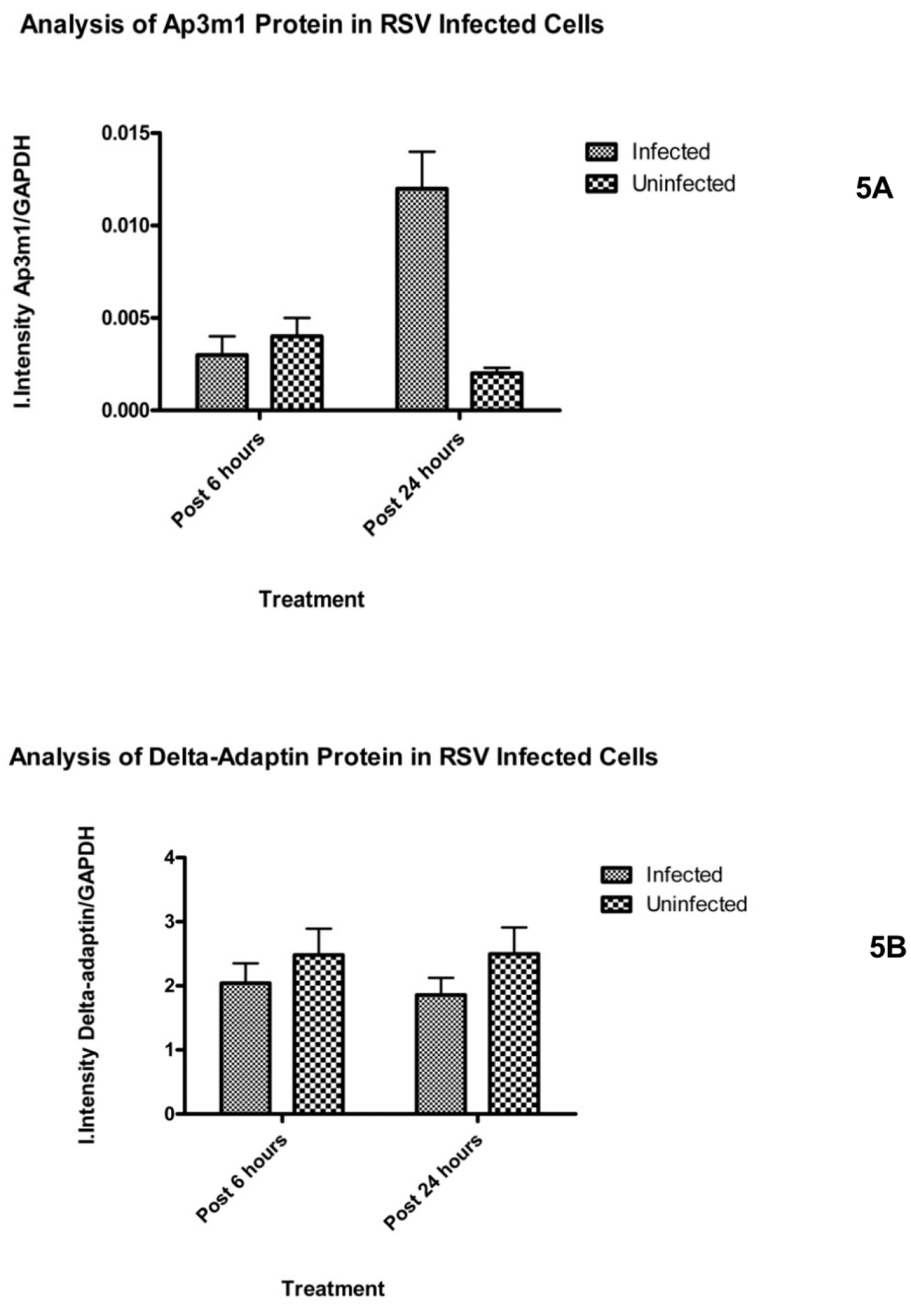
AP3Mu3A is up-regulated at 24 hours post-infection in infected cells versus mock infected HEp2 cells while at 6 hours there was no statistically significant difference. The AP3delta subunit was also analyzed and it was determined that there was no statistical significant difference in infected cells versus mock infected cells at both 6 and 24hours post-infection. 15μg of cell lysate, extracted by MPER (Pierce) were analyzed by 4–12% Bis-Tris SDS-PAGE gels (Invitrogen) in triplicate and transferred to nitrocellulose. Blots were blocked overnight in Rockland IR blocking buffer, then probed with the following antibodies diluted in 50% Tween-20 Wash Buffer: 50% Rockland blocking buffer for one hour incubation periods with extensive wash periods between incubations: Primary antibodies used are Goat anti-Ap3m1 (1:200), Mouse anti-RSV Matrix (1:1000), Rabbit anti-delta adaptin (SA4) (1:1000), Rabbit anti-GAPDH (1:10000), and secondary antibody used are Donkey anti-Goat IR 800 (1:25000), Donkey anti-Rabbit IR 800 (1:25000), Donkey anti-Rabbit IR 700 (1:5000), Donkey anti-Mouse IR 700 (1:5000), Donkey anti-Mouse IR 800 (1:20000). Blots were analyzed on Licor Imager and integral intensities were measured for each band corresponding to the 41kDa GAPDH, 47 kDa AP3Mu3A, and 160 kDa delta-adaptin bands. The statistical analysis was performed on the ratio of the integral intensity measurement of the triplicate average of the protein of interest versus the GAPDH control in infected cells versus mock-infected cells at various time points post RSV infection.

One of the interesting qualitative observations noticed throughout CLSM analysis was during late infection; there seemed to be a significant difference in the amount of AP3Mu3A seen in infected cells versus uninfected cells. To understand this, HEp2 cells were either infected or mock-infected and cell lysates were extracted at 6 and 24 hour post infection time points to be analyzed by Western Blot (Fig 5A). Equivalent amounts of cell lysate were loaded and after subsequent transfer of SDS-PAGE gels to nitrocellulose followed by probing with primary and secondary antibodies, as described in methods section; fluorescent intensities were collected on Licor Imager. Statistical analysis showed that at 6 hours there was no statistical difference between the amount of AP3Mu3A in infected versus uninfected cells (p-value = 0.5032), while at 24 hours post infection there existed a statistically significant, greater than two fold, increase in the amount of AP3Mu3A in infected cells versus uninfected cells (p-value = .0094) (Fig 5A). To determine if the whole AP-3 adaptor complex was up-regulated, the delta-adaptin subunit was also analyzed via the same protocol as the MU subunit just described. The results of this analysis showed that there was no statistically significant difference between infected and uninfected cells at 6 (p-value = 0.4415), and 24-hour post infection (p-value = .2635) (Fig 5B). This data seems to suggest that the AP-3 MU subunit is preferentially up-regulated during HRSV infection, possibly due to the requirement of the AP3Mu3A subunit for proper function of the RSV M protein during HRSV infection.

## Discussion

In this study, we show the elucidation of a novel interaction between the AP-3 complex and the HRSV M protein specifically through the AP-3Mu3A subunit, which plays a role in trafficking. This interaction was initially found via a yeast-two hybrid system and then further confirmed in mammalian models by colocalization and co-immunoprecipitation studies. Sequence alignment performed using Clustal W comparing the various RSV matrix proteins show that a highly conserved homologous binding motif, YXXL was present, providing further evidence that an interaction with the adaptor protein Mu subunits is critical for RSV to maintain function in pathogenesis across various RSV strains. The presence of a conserved tyrosine based sorting signal motif, 197-YXXL-200, across various RSV-related strains and the results of point-mutated colocalization and co-immunoprecipitation studies indicate that AP-3Mu3A mediated trafficking of M is contingent on the presence of the tyrosine and leucine residues at amino acid residue 197 and 200, respectively in the 197-YXXL-200 sorting sequence of M. The M protein in the point mutation constructs, Y-197-A and L200A Opt.M, were seen localized exclusively in nuclear and juxtanuclear regions especially absent in cytoplasmic regions extending to the cell margin (Fig 4C). The HRSV may be seen budding but appear stuck and unable to get released from the cell membrane. This observation suggests that because of the mutation of these vital tyrosine and leucine residues, the ability of AP-3 to direct M properly away from the juxtanuclear region toward cytoplasmic inclusions and/or sites of viral assembly late in infection was halted or impaired. The viral titers from mutant M protein decreased at least by 2–3 logs compared to wt-M or HRSV alone (Fig 4D). One reason for only 2–3 logs decrease in viral titer may be because the normal matrix protein produced by the virus may compete with the transfected mutant M protein and the reduction of viral titer may be due to mutant M protein incorporation into viral assembly and budding. The virus stuck on the cell membrane may not have been released into the media supernatant and this may account for reduction of viral titers in the mutant cell group. In order to get significant reduction of viral titers and the effect of virus infection, a recombinant HRSV with mutation of M in the viral genome is required which we plan to conduct future experiments with experts in the generation of recombinant HRSV.

Previous studies have elucidated various sites in the trafficking itinerary of the Matrix protein as it has been shown to exit the nucleus through a Crm-1 mediated nuclear export mechanism [7] and subsequently localize late in infection to cytoplasmic inclusions with the N, P and M2-1 proteins [14], with G in the Golgi [22] and later with the F protein at the plasma membrane in lipid rafts [5]. Recently the elucidation of a novel budding pathway by Crowe et al demonstrated that HRSV uses a budding mechanism controlled by Rab11-FIP2, a major apical recycling endosome (ARE) protein [23]. The ARE is located just below the apical membrane and is marked by the presence of Rab11a [24]. The ARE serves as a slow recycling endosome as well as the final destination for basolateral to apical membrane transcytosing proteins [25].

This has major implications in the elucidation of the trafficking itineraries of HRSV viral proteins as it has also been shown that the normal apical sorting signals, glycophosphatidylinositol (GPI) anchors, *N*-glycans, *O*-glycans, that are found only in the HRSV envelope glycoproteins (F, G and SH proteins), are not required for apical maturation or release of the virus. In addition, the deletion of one or more of the glycoprotein genes had no affect on the recruitment of the remaining viral glycoproteins or the nucleocapsid protein to the apical membrane [26]. Thus, a mechanism for the apical and basolateral trafficking of the M protein, a non-glycosylated inner virion protein, is crucial in understanding how HRSV proteins assemble at the apical surface of polarized epithelial cells.

Further insights into how the interaction between HRSV M and AP-3 may be involved in the assembly of HRSV particles late in infection stems from previous studies showing how the AP-3 complex regulates the trafficking of exogenous viral proteins within endosomal and *trans*-Golgi compartments. Studies have demonstrated the role of AP-3delta in directing the intracellular trafficking of HIV-1 Gag and for efficient transport of Vesicular Stomatitis Virus (VSV) G protein from the trans-Golgi network (TGN) to the cell-surface [27, 28, 29]. Furthermore, the di-leucine mediated interaction between the HIV-1 Nef protein and AP-1MU and AP-3Mu stabilizes the association of the AP complexes with membranes that influences the viral infectivity of HIV-1 through alteration of the early/recycling endosomal compartments [16, 17, 27, 30].

Of note, in addition to the 197-YXXL-200 basolateral sorting signal at amino acids 197–200 of the M protein, there exists three consecutive encoded leucines at amino acid residues 200–202 that could possibly make up two separate di-leucine motifs, another conserved sorting signal shown previously to regulate the interaction between adaptor protein complexes and exogenous viral proteins. Previously, in the investigation of the Crm-1 nuclear export mechanism used by the HRSV Matrix protein, Ghildyal et al disrupted the putative C-terminal nuclear export signal (cNES) at amino acids 194–206, by mutating a classic leucine rich motif. The authors mutated all three leucine residues to alanine residues and it resulted in the inability of the Matrix protein to export from the nucleus. Thus, it is difficult to assess whether or not the di-leucine motif could also be implicated as a trafficking motif because the M protein is maintained within the nucleus [7]. Recent publication has shown that the Thr205 Phosphorylation Site within matrix Protein Modulates M Oligomerization and Virus Production [31].

The observation that the AP-3Mu1 subunit is up-regulated at 24 hours post infection but not at 6 hours is also further evidence that the interaction with the AP-3 complex may be involved in viral assembly or budding. The finding that the AP-3delta subunit is not up-regulated indicates that there may be a requirement for the cells to either maintain AP-3Mu1 protein from degradation or is up-regulated at a transcriptional level.

**Fig 6:**
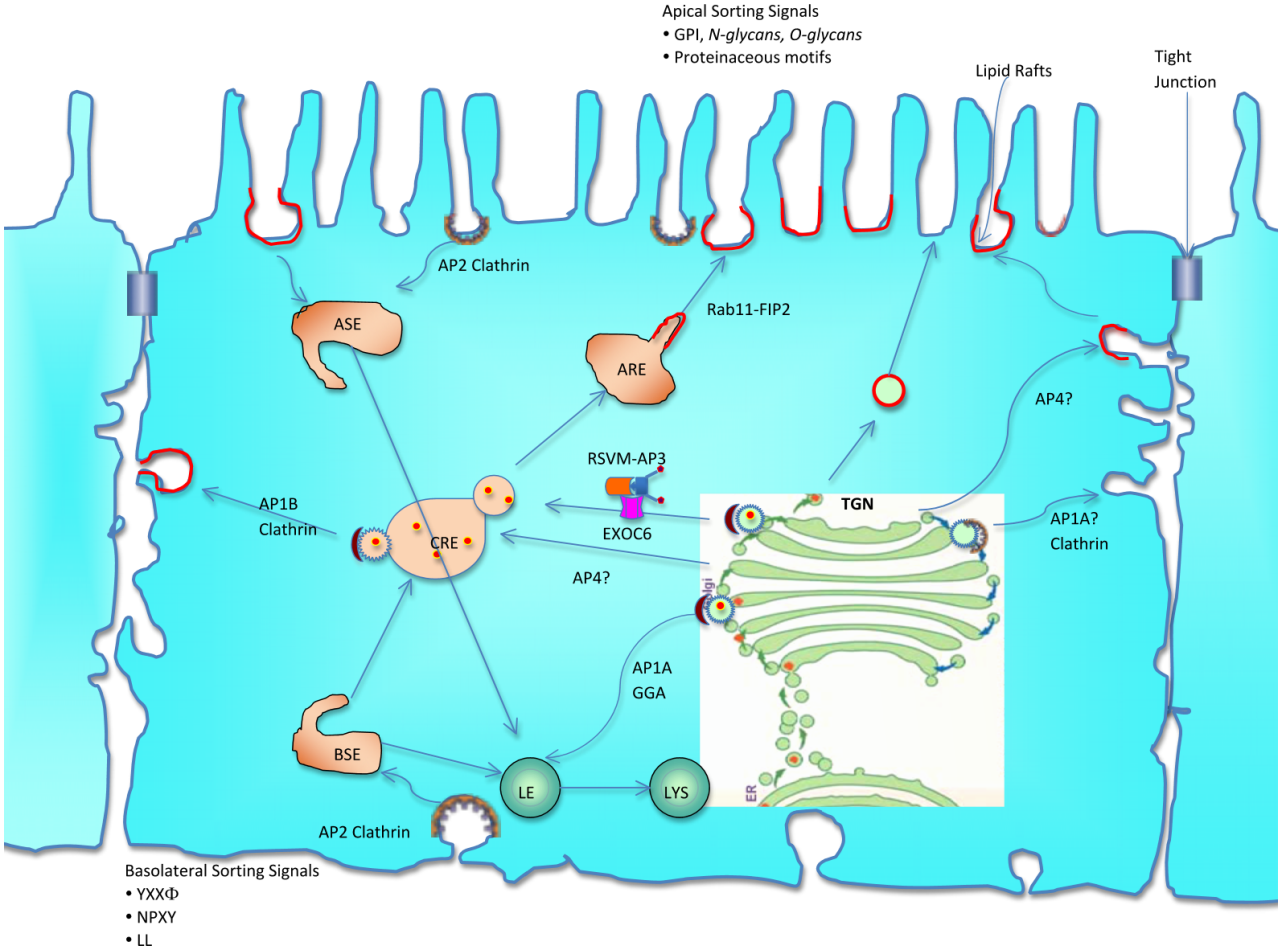
Trafficking route of HRSV M and AP-3 in epithelial cells (adapted with permission from Rodriguez-Boulan et al Nature Reviews 2005, 233–247). HRSV M protein after exiting the *trans*-Golgi network (TGN) interacts with AP3 adaptor protein via YXXL domain and reach common recycling endosomes (CREs) and apical recycling endosomes (AREs). The ARE serves as a slow recycling endosome as well as the final destination for basolateral to apical membrane transcytosing proteins. M may also interact with Exocyst Complex Component 6 (EXOC6), which is involved in vesicular trafficking from the Golgi to plasma membrane. The M protein then interacts with the major ARE-associated protein, Rab11 family interacting protein 2 (FIP2) and help in the budding of the virus through lipid rafts. Abbreviations: basal sorting endosomes (BSEs), late endosomes (LEs) and lysosomes (LYS), apical sorting endosomes (ASEs), common recycling endosomes (CREs), apical recycling endosomes (AREs), *trans*-Golgi network (TGN), Exocyst Complex Component 6 (EXOC6).

Based on our results and information from published literature, we propose a model for ‘Trafficking route of HRSV M and AP-3 in epithelial cells’, as shown in Fig 6 which was adapted with permission from Rodriguez-Boulan et al., 2005 [32]. HRSV M protein after exiting the *trans*-Golgi network (TGN) interacts with AP3 adaptor protein via YXXL domain and reach common recycling endosomes (CREs) and apical recycling endosomes (AREs). The ARE serves as a slow recycling endosome as well as the final destination for basolateral to apical membrane transcytosing proteins. A recent paper by Kipper et al., 2015 has shown that M interacts with Exocyst Complex Component 6 (EXOC6), which is involved in vesicular trafficking from the Golgi to plasma membrane [33]. The M protein may then interact with the major ARE-associated protein, Rab11 family interacting protein 2 (FIP2) [23] and help in the budding of the virus through lipid rafts.

The elucidation of the interaction between RSV M and AP-3 sheds light on one piece of a complex pathway by which viral and cellular components interact to direct RSV viral particle formation. Future research may unravel important details of the virus-host machinery that is involved in Golgi and endosomal sorting, including the roles of lipid rafts, sorting signals, adaptor proteins, and the microtubule- and actin-based cytoskeleton.

Although HRSV is a prime candidate for early childhood immunization and antiviral drug therapy, at present, the only treatment available is a monoclonal antibody administered to high-risk infants [34]. Thus, the results of this study add new insights and targets that may lead to the development of potential antivirals and attenuating mutations suitable for candidate vaccines in the future.

## Supporting information

S1 Fig The HRSV M protein co-immunoprecipitates with the AP-3Mu3A complex during HRSV infection. **a1)** The HRSV M protein co-immunoprecipitates with the AP-3Mu3A complex during HRSV infection. HEp2 cells at approximately 90% confluency were either infected at an MOI of 5 or mock infected for 24 hours, cells were scraped or proteins were subsequently extracted using MPER. Cell lysates were incubated for 6 hours with 1 μg of polyclonal goat anti-AP-3Mu3A at 4°C on a rotating device. 20μl Protein A/G agarose beads were added to lysate plus corresponding antibody and incubated overnight. Immunoprecipate complex was pelleted and washed with PBS and then ran out on a SDS-PAGE gel and transferred to nitrocellulose membrane. Membrane was blocked and then probed with monoclonal mouse anti-Matrix primary antibody as described previously for one hour. Membranes were then washed with a PBS-Tween20 solution extensively and then probed with species-specific secondary antibodies donkey anti-mouse IR dye 700. Membranes were again washed extensively and blots were imaged on Odyssey Infrared imager. The last lane shows protein molecular weight marker (KDa). The results were reproducible in at least two independent assays. **a2)** The HRSV M protein co-immunoprecipitates with the AP-3Mu3A complex during HRSV infection. HEp2 cells at approximately 90% confluency were either infected at an MOI of 5 or mock infected for 24 hours, cells were scraped or proteins were subsequently extracted using MPER. Cell lysates were incubated for 6 hours with 1 μg of polyclonal goat anti-AP-3Mu3A at 4°C on a rotating device. 20μl Protein A/G agarose beads were added to lysate plus corresponding antibody and incubated overnight. Immunoprecipate complex was pelleted and washed with PBS and then ran out on a SDS-PAGE gel and transferred to nitrocellulose membrane. Membrane was blocked and then probed with polyclonal goat-anti HRSV primary antibody as described previously for one hour. Membranes were then washed with a PBS-Tween20 solution extensively and then probed with species-specific secondary antibodies donkey anti-goat IR dye 800. Membranes were again washed extensively and blots were imaged on Odyssey Infrared imager. The last lane shows protein molecular weight marker (KDa). The results were reproducible in at least two independent assays. **b1)** The HRSV M protein co-immunoprecipitates with the AP-3delta complex during HRSV infection. HEp2 cells at approximately 90% confluency were either infected at an MOI of 5 or mock infected for 24 hours, cells were scraped or proteins were subsequently extracted using MPER. Cell lysates were incubated for 6 hours with 1 μg of monoclonal mouse anti-AP-3delta at 4°C on a rotating device. 20μl Protein A/G agarose beads were added to lysate plus corresponding antibody and incubated overnight. Immunoprecipate complex was pelleted and washed with PBS and then ran out on a SDS-PAGE gel and transferred to nitrocellulose membrane. Membrane was blocked and then probed with monoclonal mouse anti-Matrix primary antibody as described previously for one hour. Membranes were then washed with a PBS-Tween20 solution extensively and then probed with species-specific secondary antibodies donkey anti-mouse IR dye 700. Membranes were again washed extensively and blots were imaged on Odyssey Infrared imager. The last lane shows protein molecular weight marker (KDa). The results were reproducible in at least two independent assays. **b2)** The HRSV M protein co-immunoprecipitates with the AP-3delta complex during HRSV infection. HEp2 cells at approximately 90% confluency were either infected at an MOI of 5 or mock infected for 24 hours, cells were scraped or proteins were subsequently extracted using MPER. Cell lysates were incubated for 6 hours with 1 μg of monoclonal mouse anti-AP-3delta at 4°C on a rotating device. 20μl Protein A/G agarose beads were added to lysate plus corresponding antibody and incubated overnight. Immunoprecipate complex was pelleted and washed with PBS and then ran out on a SDS-PAGE gel and transferred to nitrocellulose membrane. Membrane was blocked and then probed with polyclonal goat-anti HRSV primary antibody as described previously for one hour. Membranes were then washed with a PBS-Tween20 solution extensively and then probed with species-specific secondary antibodies donkey anti-goat IR dye 800. Membranes were again washed extensively and blots were imaged on Odyssey Infrared imager. The last lane shows protein molecular weight marker (KDa). The results were reproducible in at least two independent assays. **c) Representative**. The HRSV M protein co-immunoprecipitates with the AP-3Mu3A (lanes 1–3) and AP-3delta (lanes 7–9) complex during HRSV infection. HEp2 cells at approximately 90% confluency were either infected at an MOI of 5 (lanes 1–3 and 7–9) or mock infected (lanes 4–6 and 10–12) for 24 hours, cells were scraped or proteins were subsequently extracted using MPER. Cell lysates were incubated for 6 hours with 1 μg of either polyclonal goat anti-AP-3Mu3A (lanes 1–6) or monoclonal mouse anti-AP-3delta (lanes 7–12) at 4°C on a rotating device. 20μl Protein A/G agarose beads were added to lysate plus corresponding antibody and incubated overnight. Immunoprecipate complex was pelleted and washed with PBS and then ran out on a SDS-PAGE gel and transferred to nitrocellulose membrane. Membrane was blocked and then probed with polyclonal goat-anti HRSV primary antibody as described previously for one hour. Membranes were then washed with a PBS-Tween20 solution extensively and then probed with species-specific secondary antibodies donkey anti-goat IR dye 800. Membranes were again washed extensively and blots were imaged on Odyssey Infrared imager. The samples were run in triplicates. Lane 13 shows protein molecular weight marker (KDa). The results were reproducible in at least two independent assays. **d) Representative negative control**. HEp2 cells at approximately 90% confluency were either infected at an MOI of 5 (lanes 1–3 and 7–9) or mock infected (lanes 4–6 and 10–12) for 24 hours, cells were scraped or proteins were subsequently extracted using MPER. Cell lysates were incubated for 6 hours with 1 μg of either polyclonal goat anti-H1N1antibodies (lanes 1–6) or monoclonal mouse anti-HIV-1 p24 Gag antibodies (lanes 7–12) at 4°C on a rotating device. 20μl Protein A/G agarose beads were added to lysate plus corresponding antibody and incubated overnight. Immunoprecipate complex was pelleted and washed with PBS and then ran out on a SDS-PAGE gel and transferred to nitrocellulose membrane. Membrane was blocked and then probed with polyclonal goat-anti HRSV primary antibody as described previously for one hour. Membranes were then washed with a PBS-Tween20 solution extensively and then probed with species-specific secondary antibodies donkey anti-goat IR dye 800. Membranes were again washed extensively and blots were imaged on Odyssey Infrared imager. The samples were run in triplicates. Lane 13 shows protein molecular weight marker (KDa). The results were reproducible in at least two independent assays.
(TIF)
