# Supplementary figures and images for: Interaction of the Human Respiratory Syncytial Virus matrix protein with cellular adaptor protein complex 3 plays a critical role in trafficking
Source: PLoS One. 2017 Oct 13;12(10):e0184629. doi: 10.1371/journal.pone.0184629 (PMC5640227; doi:10.1371/journal.pone.0184629)

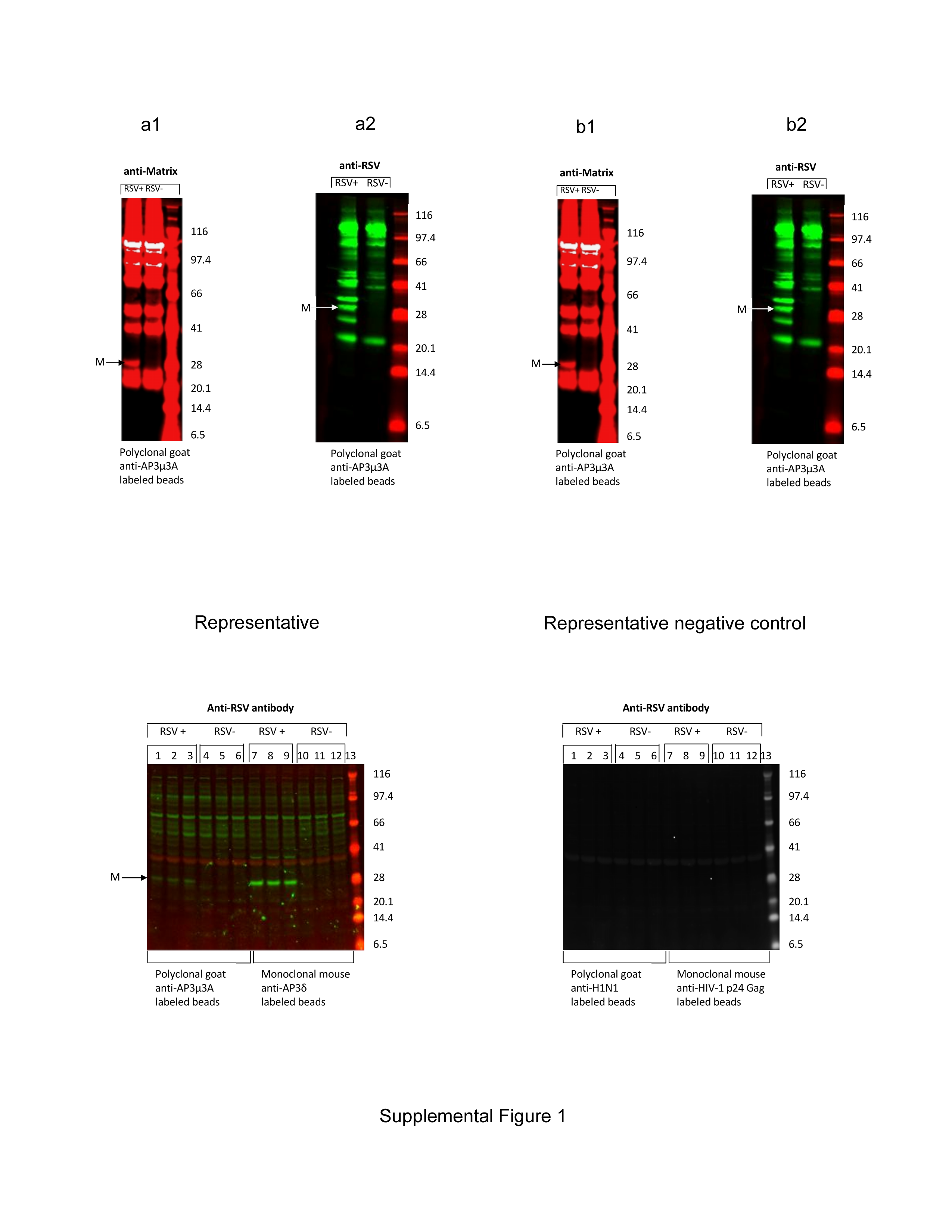

Supplement: S1 Fig — a1) The HRSV M protein co-immunoprecipitates with the AP-3Mu3A complex during HRSV infection. HEp2 cells at approximately 90% confluency were either infected at an MOI of 5 or mock infected for 24 hours, cells were scraped or proteins were subsequently extracted using MPER. Cell lysates were incubated for 6 hours with 1 μg of polyclonal goat anti-AP-3Mu3A at 4°C on a rotating device. 20μl Protein A/G agarose beads were added to lysate plus corresponding antibody and incubated overnight. Immunoprecipate complex was pelleted and washed with PBS and then ran out on a SDS-PAGE gel and transferred to nitrocellulose membrane. Membrane was blocked and then probed with monoclonal mouse anti-Matrix primary antibody as described previously for one hour. Membranes were then washed with a PBS-Tween20 solution extensively and then probed with species-specific secondary antibodies donkey anti-mouse IR dye 700. Membranes were again washed extensively and blots were imaged on Odyssey Infrared imager. The last lane shows protein molecular weight marker (KDa). The results were reproducible in at least two independent assays. a2) The HRSV M protein co-immunoprecipitates with the AP-3Mu3A complex during HRSV infection. HEp2 cells at approximately 90% confluency were either infected at an MOI of 5 or mock infected for 24 hours, cells were scraped or proteins were subsequently extracted using MPER. Cell lysates were incubated for 6 hours with 1 μg of polyclonal goat anti-AP-3Mu3A at 4°C on a rotating device. 20μl Protein A/G agarose beads were added to lysate plus corresponding antibody and incubated overnight. Immunoprecipate complex was pelleted and washed with PBS and then ran out on a SDS-PAGE gel and transferred to nitrocellulose membrane. Membrane was blocked and then probed with polyclonal goat-anti HRSV primary antibody as described previously for one hour. Membranes were then washed with a PBS-Tween20 solution extensively and then probed with species-specific secondary antibodie [file pone.0184629.s001.tif]
